# *In Vitro* Toxicity Evaluation of Engineered Cadmium-Coated Silica Nanoparticles on Human Pulmonary Cells

**DOI:** 10.1155/2013/931785

**Published:** 2013-09-30

**Authors:** Uliana De Simone, Luigi Manzo, Antonella Profumo, Teresa Coccini

**Affiliations:** ^1^Department of Clinical-Surgical, Diagnostic and Pediatric Sciences, University of Pavia, 27100 Pavia, Italy; ^2^Laboratory of Clinical Toxicology, IRCCS Maugeri Foundation, Medical Institute of Pavia, 27100 Pavia, Italy; ^3^Department of Chemistry, University of Pavia, 27100 Pavia, Italy

## Abstract

Cytotoxicity of cadmium-containing silica nanoparticles Cd-SiO_2_NPs (0.05–100 *µ*g/mL) versus SiO_2_NPs and CdCl_2_ was evaluated by an *in vitro* test battery in A549 by assessing (i) mitochondrial function, (ii) membrane integrity/cell morphology, (iii) cell growth/proliferation, (iv) apoptotic pathway, (v) oxidative stress, after short- (24–48 h) and long-term (10 days) exposure. Both Cd-SiO_2_NPs and CdCl_2_ produced dose-dependent cytotoxic effects: (i) *MTT-assay*: similar cytotoxicity pattern was observed at both 24 and 48 h, with a more Cd-SiO_2_NPs pronounced effect than CdCl_2_. Cd-SiO_2_NPs induced mortality (about 50%) at 1 **μ**g/mL, CdCl_2_ at 25 **μ**g/mL; (ii) *calcein-AM/PI staining*: decrease in cell viability, noticeable at 25 **μ**g/mL, enhanced markedly at 50 and 100 **μ**g/mL, after 24 h. Cd-SiO_2_NPs induced higher mortality than CdCl_2_ (25% versus 4%, resp., at 25 **μ**g/mL) with further exacerbation after 48h; (iii) *clonogenic assay*: exposure for longer period (10 days) compromised the A549 proliferative capacity at very low dose (0.05 **μ**g/mL); (iv) a progressive activation of *caspase-3 immunolabelling* was detected already at 1 **μ**g/mL; (v) GSH intracellular level was modified by all compounds. In summary, *in vitro* data demonstrated that both Cd-SiO_2_NPs and CdCl_2_ affected all investigated endpoints, more markedly after Cd-SiO_2_NPs, while SiO_2_NPs influenced GSH only.

## 1. Introduction

The rapid development of nanotechnology worldwide is accompanied by massive generation and usage of engineered nanoparticles (ENPs), even though essentially most of these NPs have not been sufficiently examined for potential toxicity at this time [1, 2]. Thus, with the exponential growing production of ENPs, the potential for the respiratory system to be exposed to a seemingly countless number of unique NPs is expected to increase, and many aspects related to the size of these nanomaterials, smaller than cells and cellular organelles, have raised concerns about safety [2–4].

Among ENPs, silica/cadmium containing nanomaterials have attracted much attention in the latest years for their applications in medicine and industrial manufacturing, synthesis, and engineering [5–10]. Though silica nanoparticles (SiO_2_NPs) are generally considered to be nontoxic, experiments using cell cultures or animal models have indicated dose-dependent cytotoxicity, increased reactive oxygen species, and reversible lung inflammation [11–19]. On the other hand, a large body of evidence supports lung toxicity effects after cadmium exposure when inhaled [20, 21], and although its toxicity mechanisms are not yet fully understood, several reports have described pulmonary inflammatory changes and induction of oxidative stress in response to cadmium inhalation exposure [22]. 

Some NPs, such as metal-based nanomaterials should represent risk factors for lung diseases, as many of these metals in their native form are known to have fibrogenic, inflammogenic or carcinogenic effects in humans. Evaluation of the NPs biosafety is essentially required by tests examining general toxicity, target organ toxicity, and biocompatibility in line with regulatory requirements and to identify molecular endpoints and multiple toxicity pathways.

The present study intended to elucidate the toxicological profile of a model nanomaterial namely cadmium-containing silica nanoparticles (Cd-SiO_2_NPs) by an *in vitro* testing approach. 

For instance, a key concept, developed from the strategy proposed by major institutions [23, 24] and international consensus meetings [25], indicates the use of multi-tiered testing protocols to address toxicological research and health risk assessment for NPs, based on (i) physic-chemical characterization, (ii) *in vitro* models by a battery of cytotoxicity tests, and (iii) *in vivo* experiments driven on the basis of the *in vitro* results. Information generated using *in vivo* studies will also provide a database from which to compare *in vitro* studies for identifying additional evidence that assists in explaining findings from *in vivo* nanomaterial toxicity or health effects. Comparing the *in vitro* and *in vivo* results may also help to assess the concordance/discordance between the alternative methods and the *in vivo* methods, and to test the predictability of the alternative methods for the *in vivo* results [26].

The identification of predictive *in vitro* toxicity assays is in line with the recommended attention that should be given to regulatory acceptance as means of promoting the use of alternative methods to animal testing in human safety assessment [27, 28]. 

Our recent *in vivo* investigation on Cd-SiO_2_NPs indicated long-lasting lung damage, after intratracheal instillation (i.t.) of these nanoparticles in rats, characterized by morphological alterations, the occurrence of inflammation (accompanied by granuloma formation), stromal fibrogenic reaction, and enhancement of apoptotic phenomena followed by a consequent increased cell proliferation [29]. This pulmonary insult was also associated with an oxidative stress response [30]. 

In this work, a battery of *in vitro* tests have been used to examine the responses of human lung epithelial cells to Cd-SiO_2_NPs exposure in terms of metabolic activity (by MTT assay), membrane integrity (by calcein-AM/Propidium Iodide staining), oxidative stress (by GSH content evaluation), apoptosis (by activated caspase-3 evaluation), and growth and cell proliferation (by clonogenic assay) to determine whether the modification of morphological and biochemical parameters evaluated by *in vitro* investigations are in accordance with *in vivo* pulmonary insult caused by these nanoparticles. The effects of Cd-SiO_2_NPs were assessed in A549 cell line, which represents a widely used cell model to investigate alveolar cell function [31], after short- (24–48 hours) and long-term (10 days) exposure and compared with those caused by treatments with cadmium chloride (CdCl_2_) and SiO_2_NPs.

## 2. Materials and Methods

### 2.1. Chemicals

All cell culture reagents, chemicals, and cadmium chloride hemi (pentahydrate; CdCl_2_) were obtained from Sigma-Aldrich (Milan, Italy). Caspase 3 and Alexa 488-labeled antibodies from Molecular Probes (Life Technologies, Monza, Italy) and the GSH quantification Kit from Oxis International Inc. (Foster City, Ca, USA). Silica nanosize (SiO_2_NP) was purchased from Degussa Gmbh (Germany) as HiSilTM T700, average pore size 20 nm, surface area 240 m^2^/g, and pore specific volume of 0.4 cm^3^/g.

### 2.2. Synthesis and Physico-Chemical Characterization of Engineered Cadmium-Coating Nanoparticles (Cd-SiO_2_NPs)

Synthesis and physico-chemical characterization of Cd-SiNPs are previously described in Coccini et al. [29]. Briefly, Cd-SiO_2_NPs were produced by the impregnation of SiO_2_NPs with cadmium nitrate dehydrate (CdNO_3_ 3.56 × 10^−2^ M) in an aqueous solution with silica dispersed in a concentration ratio leading to a sample containing 40% Cd by weight. Powder was later subjected to grinding mills with high energy (200 rpm for 1.5 h, 400 rpm for 1.5 h, 600 rpm for 2 h) to get the most equal distribution of particle size and/or aggregates of particles. The entire synthesis preparation was performed under sterile condition to avoid NPs contamination. 

Quantitative analyses by scanning transmission electron microscopy (STEM) showed the aggregation of Cd-SiO_2_NPs and the analysis of the elements in High Angle Annular Dark Field (HAADF) mode (energy-dispersive (EDS) spectra) confirmed the presence of Cd, Si, and O. X-ray diffraction demonstrated amorphous and crystalline phases of the sample. Dynamic light scattering (DLS) of the Cd-SiO_2_NPs showed tendency to form aggregates and agglomerates of about 350 nm (zeta potential about −23 mV in DMEM). Particles presented spherical form, primary particle size range of 20–80 nm and specific surface area of about 200 m^2^/g. Metal impurities (Ca (0.3%), Na (0.2%), K (0.2%), Fe (0.04%), and Mn (0.001%)) and the release of cadmium from nanoparticles dispersed in culture medium were determined by flame-atomic absorption analysis. Maximum cadmium release in culture medium (DMEM) was 28% after 16 h, and it was negligible in the subsequent 10-day period.

### 2.3. Cell Line and Cell Culture

Human lung epithelial cells (A549 cell line purchased from ECACC, Sigma-Aldrich, Milan, Italy) were used for *in vitro* study of the nanoparticle toxicity. Cells were cultured in DMEM supplemented with 10% fetal bovine serum (FBS), 2 mM L-glutamine, 50 IU/mL penicillin, and 50 *μ*g/mL streptomycin. Cells were maintained at 37°C in a humidified atmosphere (95% air/5% CO_2_).

Stock solutions were prepared by dissolving test materials (Cd-SiO_2_NPs, CdCl_2_, and SiO_2_NPs) in culture medium (DMEM), then cells were exposed to concentrations ranging from 0.05 to 100 *μ*g/mL. Fresh solutions of test materials were prepared shortly before each experiment.

Doses were chosen based on previous experiments in A549 cells showing toxic effects (e.g., apoptosis, necrosis) after cell exposure to concentrations ranging from 5 to 60 *μ*M (corresponding: 0.916 to 10.99 *μ*g/mL) of CdCl_2_ [32–35].

### 2.4. Cytotoxicity Study: Short-Term Exposure (24–48 h)

#### 2.4.1. Mitochondrial Function (MTT Assay) and Membrane Integrity (Calcein-AM/Propidium Iodide Staining)

The viability was assessed by two dye-based methods: MTT assay (mitochondrial function) and calcein-AM/Propidium Iodide (PI) staining (membrane integrity). Cells were seeded in 96-well plates at density of 1 × 10^4^ cells/well in complete medium. After 24 h of cell attachment, the cells were exposed to Cd-SiO_2_NPs at final concentration of Cd between 1 to 100 *μ*g/mL for 24 or 48 h at 37°C and compared to equivalent amount of CdCl_2_ or SiO_2_NPs. 

At the end of the incubation period, the mitochondrial function was assessed by 0.5 mg/mL MTT (3-(4,5-dimethylthiazol-2-yl)-2,5-diphenyltetrazolium bromide) for 3 h at 37°C and was quantified spectrophotometrically at 550 nm in Biorad microplate reader. Data were expressed as a percentage of control (untreated cells).

The membrane integrity was evaluated by the co-incubation of the double staining: 2 *μ*M calcein-AM and 2.5 *μ*g/mL PI for 5 min at 37°C. Cells were examined under a Zeiss Axiovert 25 fluorescence microscope combined with a digital camera (Canon powershot G8). The fluorescence images were taken using 32x objective lens with an excitation wavelength of 400, 495, 570 nm; beamsplitter wavelength of 410, 505, 585 nm; and an emission wavelength of 460, 530, 610 nm. Viability was expressed as percent cells retained calcein compared to the total cells counted (calcein-positive plus PI-positive). 

#### 2.4.2. Oxidative Stress Evaluation: Glutathione (GSH) Measurement

The concentration of intracellular GSH was determined by colorimetric assay. Briefly, cells were seeded in six-well plates at density of 5 × 10^5^ cells/well. After the treatments with 2 mL of Cd-SiO_2_NPs, CdCl_2_ and SiO_2_NPs (final concentration ranging from 1 to 100 *μ*g/mL in cell culture medium) for 24 and 48 h exposure, the medium was aspired and the cells was washed once with phosphate buffer saline (PBS). Then, the cells were scraped and centrifuged at 1100 rpm for 3 min at 25°C, the superrnatant was removed by aspiration. The cell pellets were resuspended in ice-cold metaphosphoric acid (MPA) and immediately homogenized (Ultra Turrax, Janke & Kunkel) then centrifuged at 3000 g, 4°C for 10 min. Subsequently, the samples were mixed with 4-Chloro-1-Methyl-7-Trifluromethyl-Quinolinium Methylsulfate and 30% sodium hydroxide reagents, and then were incubated for 10 min at room temperature (r.t.) in dark. The absorbance was measured spectrophotometrically (Spectrometer Lambda Bio 20, Perkin Elmer) at 400 nm, total glutathione content was determined with a standard curve.

#### 2.4.3. Apoptotic Pathway: Immunofluorescence Detection of Activated Caspase 3

Cells were seeded in coverslips at density of 2 × 10^5^ cells. After 24-h cell attachment, the cells were exposed to increasing concentrations of Cd-SiO_2_NPs, CdCl_2_, and SiO_2_NPs (1–50 *μ*g/mL) for 24 h at 37°C. At the end of incubation period, the cells were fixed with 4% paraformaldehyde for 20 min at r.t. and then in 70% ethanol over night at −20°C. After rehydration with PBS, the samples were incubated with blocking solution for 30 min at r.t., and then with polyclonal antibodies recognizing caspase 3 (dilution 1 : 200 in PBS). After washing, the bound antibodies were revealed with Alexa 488-labeled (dilution 1 : 100 in PBS) antibody recognizing rabbit-Ig. The slides were counterstained for DNA with 1 *μ*g/mL Propidium Iodide, washed with PBS and finally mounted with Fluoroshield. Cells were examined under a CX41 Olympus fluorescence microscope, excitation light being provided by EPI LED Cassette (FRAEN, Settimo Milanese (MI), Italy) and combined with digital camera (Infinity2). Digital images were captured using 100x objective lens, measurement conditions were: 470 nm excitation (*T*% = 40), 505 nm dichroic beamsplitter, 510 nm long pass filter.

### 2.5. Cytotoxicity Study: Long-Term Exposure (10 Days)

#### 2.5.1. Clonogenic Assay

The procedure for clonogenic assay was adopted from Herzog et al. [36]. A549 cells were seeded in six-well at density of 400 cells/well, each well containing 2 mL of cell culture medium. After attachment (about 14 h, time was shorter than the population doubling time), the cells were washed with 2 mL PBS, and treated with 2 mL of Cd-SiO_2_NPs, CdCl_2_, and SiO_2_NPs (final concentration ranging from 0.05 to 100 *μ*g/mL in cell culture medium) over a time period required to form colonies (about 10 days). A colony being defined as at least 50 clones of one cell. At the end of the treatment, the medium was removed and the colonies were fixed, stained with Hematoxylin and then counted for the evaluation of cell survival after Cd-SiO_2_NP, CdCl_2_ and SiO_2_NP treatments. The colonies were examined under Zeiss Axiovert 25 microscope combined with a digital camera (Canon powershot G8).

Digital photographs were taken from each well using 2.5x objective lens. The number of colonies that arose after treatment (surviving fraction) was expressed in terms of plating efficiency (PE). PE was calculated by dividing the number of colonies formed by the number of cells plated per 100.

### 2.6. Statistics

Data from acute exposure were obtained from three independent experiments each carried out in six replicates. Data from chronic exposure were obtained from two independent experiments and each experiment was carried out in three replicates. Results are expressed as mean ± SD. Statistical significance was assessed by one-way ANOVA. A value of *P* < 0.05 was considered statistically significant.

## 3. Results

### 3.1. Cytotoxic Activity of Cd-SiO_2_NPs Compared to CdCl_2_ and SiO_2_NPs in A549 Cell Line

*In vitro* cytotoxicity results after short- (24–48 h) and long-term (10 days) exposure of A549 cells to increasing concentrations of Cd-SiO_2_NPs, CdCl_2_, and SiO_2_NPs (from 0.05 to 100 *μ*g/mL) are reported and compared. Mitochondrial function, membrane integrity, oxidative stress, apoptosis were considered as endpoints of acute exposure, while the capacity to form colonies was considered as endpoint of chronic exposure.

### 3.2. Cytotoxicity Results after Short-Term Exposure (24–48 h)

#### 3.2.1. Mitochondrial Function: MTT Assay

**Figure 1:**
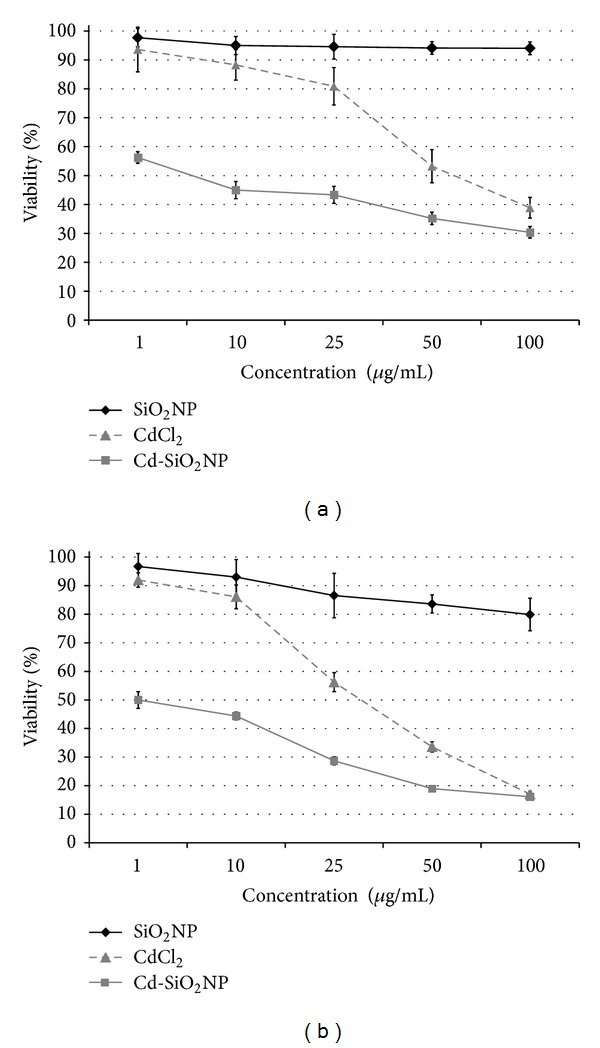
Cell viability measured by MTT assay in A549 cells exposed to increasing concentration (1–100 *μ*g/mL) of Cd-SiO_2_NPs (-□-), CdCl_2_ (-*▵*-), and SiO_2_NPs (-*◆*-) after 24 (a) and 48 h (b). Data are mean ± DS of three separate experiments each carried out in six replicates. Error bars: ±SD.

Data of mitochondrial function, evaluated by MTT after 24 and 48 h of exposure to increasing concentrations of Cd-SiO_2_NPs, CdCl_2_, or SiO_2_NPs (1–100 *μ*g/mL) and expressed as percentage of the viability of control, are presented in Figures 1(a) and 1(b). Both Cd-SiO_2_NPs and CdCl_2_ produced a dose-dependent cytotoxic effect on A549 cells. The pattern of cytotoxicity was similar at both time points (i.e., 24 and 48 h) for either compound, but Cd-SiO_2_NP cytotoxicity was more pronounced compared to CdCl_2_. Cd-SiO_2_NPs induced mortality (about 40% and 50% after 24 and 48 h exposure resp.) already at the lowest dose (1 *μ*g/mL) (Figures 1(a) and 1(b)). The cytotoxic effect of CdCl_2_ treatment was detected at 25 *μ*g/mL with about 20% mortality after 24 h (Figure 1(a)) and 45% after 48 h exposure (Figure 1(b)). The maximum effect (about 80% mortality) of the two tested materials reached at the highest dose (100 *μ*g/mL) after 48 h exposure (Figure 1(b)). 

As graphically represented, in Figures 1(a) and 1(b) (see black line), SiO_2_NPs did not show any significant cytotoxic effect after both time points considered (24 and 48 h).

#### 3.2.2. Membrane Integrity: Calcein-AM/PI Staining

**Figure 2:**
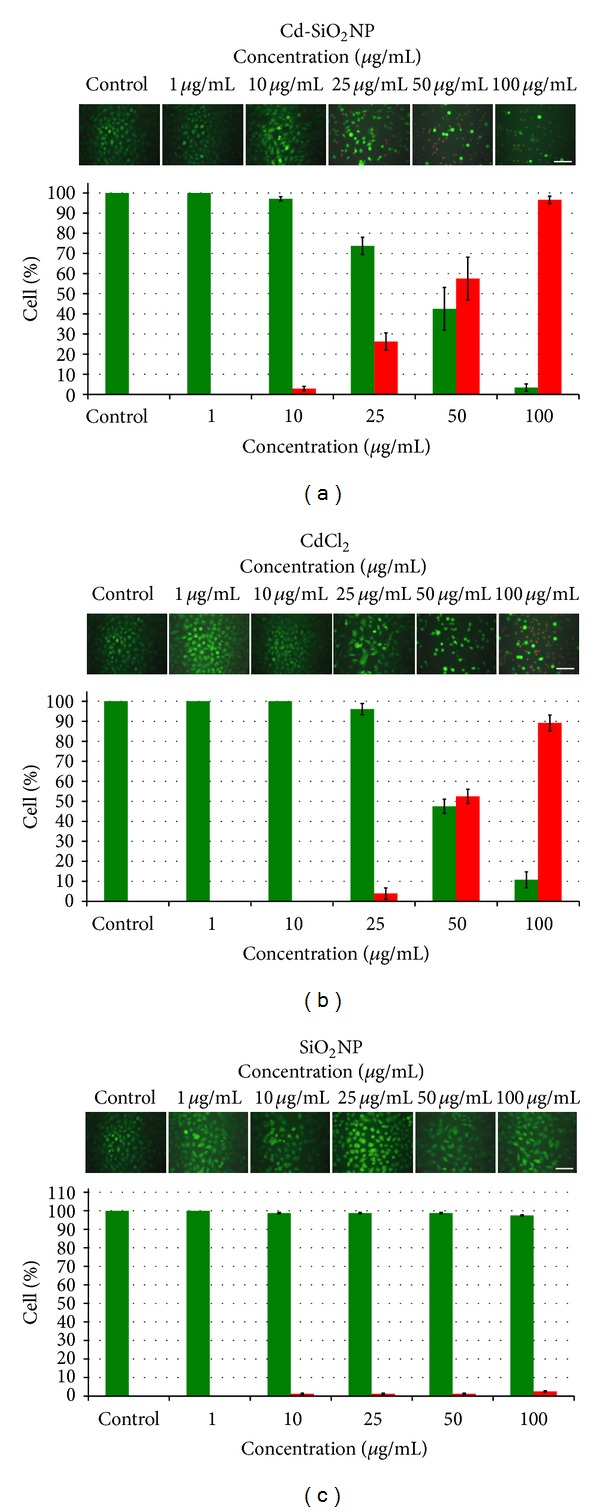
Representative images of randomly selected microscopic fields of A549 cells stained with calcein-AM/PI after 24 h exposure to increasing concentration (1–100 *μ*g/mL) of Cd-SiO_2_NPs (a), CdCl_2_ (b), and SiO_2_NPs (c). Dose-dependent cytotoxic effect in both Cd-SiO_2_NP and CdCl_2_ treatment groups: there was a strong decrease of viability at higher concentrations ranging from 50 to 100 *μ*g/mL (low or no green fluorescence and red fluorescence indicating cell death). SiO_2_NPs treated cells showed uniformly diffused green fluorescence at all tested doses similarly to cell controls. Quantitative analysis of the cell loss is shown for each treatment (Green Square Cell Live; Red Square Cell Death). Data are mean ± DS of three separate experiments, error bars: ±SD. (Scale bar: 100 *μ*m).

Membrane integrity was evaluated by calcein-AM/PI staining after 24 and 48 h exposure to the compounds. Figures 2(a), 2(b), and 2(c) describe a panel of representative and randomly selected microscopic fields of A549 cells treated with increasing concentrations of Cd-SiO_2_NPs, CdCl_2_, and SiO_2_NPs (1–100 *μ*g/mL) after 24 h.

Calcein-AM/PI staining indicated cytotoxic effects. A similar dose-dependent cytotoxic effect was observed after both Cd-SiO_2_NPs and CdCl_2_ treatments (Figures 2(a) and 2(b)). A decrease in cell viability was observed as evidenced by the presence of numerous red coloured cells (indicating damage to the cell membrane), starting at 25 *μ*g/mL dose and becoming markedly evident at the highest concentrations of 50 and 100 *μ*g/mL (Figures 2(a) and 2(b)). Semi-quantitative analysis of selected microscopic fields, after 24 h exposure, in terms of cell counts and expressed as percentage of live cells (green fluorescence), showed difference in cell death between Cd-SiO_2_NPs and CdCl_2_ treatments at dose of 25 *μ*g/mL. Cd-SiO_2_NPs mortality was higher than that caused by CdCl_2_ treatment (about 25% versus 4%). In both Cd-SiO_2_NP and CdCl_2_ groups treated with the lowest dose (1 *μ*g/mL) the green fluorescence was uniformly diffused in cell cytoplasm (indicating the maintained membrane integrity), and cell morphology was not altered (Figures 2(a) and 2(b)). 

The effects of Cd-SiO_2_NPs and CdCl_2_ were exacerbated after 48 h exposure. Cell viability was decreased of about 60–100% at doses ranging 25–100 *μ*g/mL (data not shown).

Images obtained from SiO_2_NP treatment showed uniformly diffused green fluorescence and normal cell morphology for all treatment concentrations (1–100 *μ*g/mL) when compared to control (Figure 2(c)). Semi-quantitative analysis with increasing concentrations of SiO_2_NP treatment (1–100 *μ*g/mL) showed no effects on A549 cells even at the highest dose investigated of 100 *μ*g/mL and after both 24 (Figure 2(c)) and 48 h (data not shown).

#### 3.2.3. Oxidative Stress: Evaluation of GSH Intracellular

**Figure 3:**
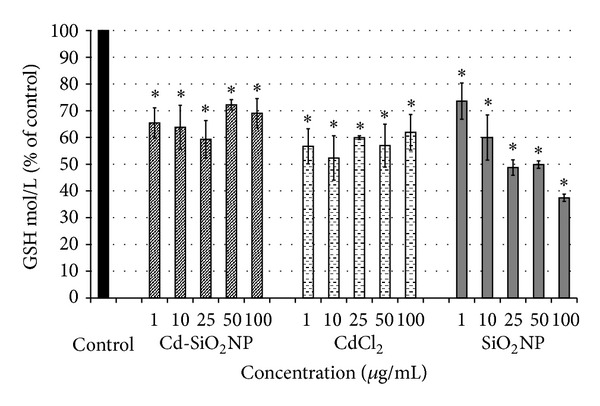
Glutathione (GSH) measurement in A549 cells unexposed (control) and exposed to increasing concentrations (1–100 *μ*g/mL) of Cd-SiO_2_NPs, CdCl_2_, SiO_2_NPs after 48 h. GSH levels were affected by all tested materials: the GSH depletion in A549 cells was not dose-dependent after Cd-SiO_2_NP or CdCl_2_ treatments, while was dose-depended after SiO_2_NP treatments. Data are mean ± DS of three separate experiments each carried out in six replicates. *P < 0.05 significant difference from control.

GSH levels were affected by all test materials at both time points considered (24–48 h). The reduction of intracellular GSH levels caused by Cd-SiO_2_NP or CdCl_2_ treatments was not dose-dependent: the GSH depletion was ranging from 35 to 40% and from 35 to 45% for Cd-SiO_2_NPs and CdCl_2,_ respectively, after 48 h exposure (Figure 3). SiO_2_NPs showed dose-depended depletion of GSH in cells when compared to control with about 55% decrease of GSH intracellular after 48 h exposure at the highest concentration investigated (100 *μ*g/mL; Figure 3). 

#### 3.2.4. Apoptotic Pathway: Immunofluorescence Analysis of Activated Caspase 3

**Figure 4:**
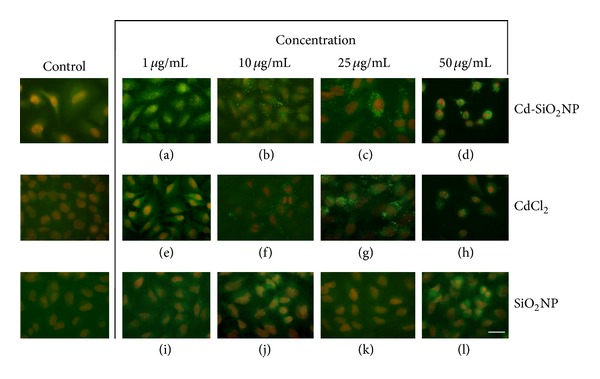
Representative images of randomly selected microscopic fields of A549 cells marked with caspase 3 antibody after 24 h incubation with: Cd-SiO_2_NPs ((a)–(d)), CdCl_2_ ((e)–(h)), SiO_2_NPs ((i)–(l)) at the doses indicated in the figure. Activation of caspase 3 is indicated by green spots already at the lowest tested dose of 1 *μ*g/mL in both Cd-SiO_2_NP and CdCl_2_ treatment groups, while there was no positivity in SiO_2_NPs treated cells at all the tested doses similarly to the unexposed cells (control). (Scale bar: 100 *μ*m).

Figure 4 displays a panel of representative randomly selected microscopic fields of A549 cells treated with increasing concentrations of Cd-SiO_2_NPs, CdCl_2_, and SiO_2_NPs (1–50 *μ*g/mL). A549 cells exposed to Cd-SiO_2_NPs or CdCl_2_ showed a progressive activation of caspase 3 as brilliant green intracellular spots (Figures 4(a)–4(d) and Figures 4(e)–4(h), resp.) directly observed with the lowest dose tested of 1 *μ*g/mL. To note, the advanced stage of apoptosis were observed at the highest dose of 50 *μ*g/mL Cd for both Cd-SiO_2_NP or CdCl_2_ treatments (Figures 4(a)–4(d) and Figures 4(e)–4(h), resp.). In addition, a decrease in cell number along with a marked alteration of the morphology can be easily appreciated. On the contrary, the immunocytochemistry analysis to detect activated caspase 3 after 24 h exposure demonstrated no positivity for cells treated with SiO_2_NPs after any tested doses (1–50 *μ*g/mL; Figures 4(i)–4(l)) and no appreciable morphology alterations compared to control.

### 3.3. Cytotoxicity Results after Long-Term Exposure (10 Days)

#### 3.3.1. Clonogenic Assay

**Figure 5:**
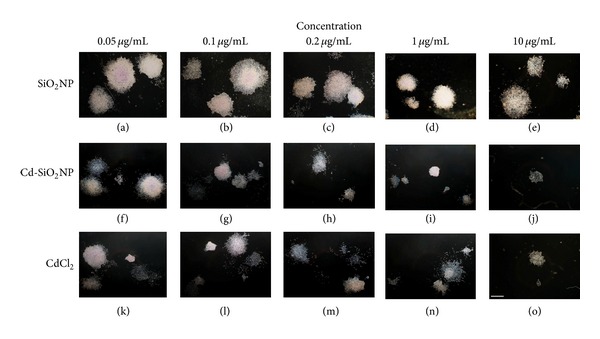
Representative images of randomly selected microscopic fields of the colonies formed after 10 consecutive days exposure to increasing concentrations (0.05–100 *μ*g/mL) of Cd-SiO_2_NPs, CdCl_2_, and SiO_2_NPs. SiO_2_NP colonies showed similar patterns at the all tested doses to the control (control data not shown). Few colonies with reduced size compared to control were observed at the lower concentrations (0.05–10 *μ*g/mL) of Cd-SiO_2_NPs and CdCl_2_, while there was a total inhibition of the colonies at the higher tested doses (25–100 *μ*g/mL) for both Cd-SiO_2_NPs and CdCl_2_. (Scale bar: 600 *μ*m).

To determine whether the prolonged exposure (up to 10 days) to increasing concentrations (0.05–100 *μ*g/mL) of Cd-SiO_2_NPs, CdCl_2_, and SiO_2_NPs might have adverse effects, the proliferation ability and colony forming capacity of A549 cells were evaluated.  Figure 5 shows representative images of randomly selected microscopic fields of the different treatment groups (Cd-SiO_2_NPs, CdCl_2_, and SiO_2_NPs). The colonies of SiO_2_NP groups (Figures 5(a)–5(e)) had roundish colony morphology and similar patterns to control (data not shown), while, A549 cells treated with doses ranging from 0.05 to 10 *μ*g/mL of Cd-SiO_2_NPs and CdCl_2_ presented few colonies and a drastic reduced size compared to control (data not shown, Figures 5(f)–5(j), and Figures 5(k)–5(o), resp.). At the higher concentrations of either Cd-SiO_2_NPs or CdCl_2_ (25 to 100 *μ*g/mL) there was a complete inhibition of the colony formation.

**Figure 6:**
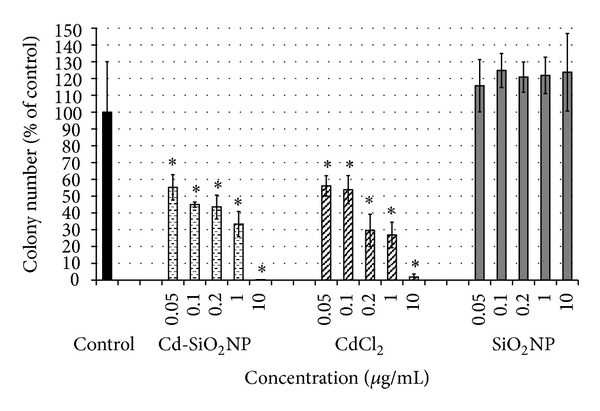
Histograms show the number of colonies formed after 10 consecutive days exposure to increasing concentrations of Cd-SiO_2_NPs, CdCl_2_, SiO_2_NPs at the doses indicated in the figure. The formation of A549 colonies appeared to be serious compromised by both Cd-SiO_2_NP and CdCl_2_ treatments already at the lowest dose of 0.05 *μ*g/mL, while SiO_2_NPs had no effects on their formation. Data are mean ± DS of two separate experiments each carried out in three replicates, expressed as percentage of control colonies. *P < 0.05 significant difference from control. Error bars: ±SD.

Semi-quantitative analysis showed a strong reduction of colony number (about 45%) already at the lowest tested dose (0.05 *μ*g/mL) for both Cd-SiO_2_NPs and CdCl_2_ treatments, until reaching a total inhibition of cell proliferation at the doses from 25 to 100 *μ*g/mL (Figure 6). SiO_2_NP treatment did not produce adverse effects, indeed the proliferative activity of A549 cells was not inhibited after prolonged exposure to any SiO_2_NP dose tested (0.05–100 *μ*g/mL; Figure 6). 

## 4. Discussion

The present *in vitro* investigations indicate that Cd-SiO_2_NP treatment produced *in vitro* deleterious effects after short- (24–48 h) and long-term (up to 10 days) exposure with mitochondrial function severely impaired and activation of caspase-3, depletion of GSH, and inhibition of cell proliferation already observable at the lowest concentration doses (i.e., 1 *μ*g/mL for the short-term exposure and 0.05 *μ*g/mL for the prolonged exposure). Similar cytotoxic profile was observed after CdCl_2_ treatment. However, the magnitude of effects caused by Cd-SiO_2_NPs was more pronounced compared to that produced by CdCl_2_. 

A549 acutely exposed to Cd-SiO_2_NPs showed dose-dependent alterations of mitochondrial function and membrane integrity, as well as activation of caspase-3 already after 24 h exposure. At 48-hr, a further exacerbation of these effects was assessed. CdCl_2_ also influenced the same cellular parameters although the effects were less pronounced than those caused by Cd-SiO_2_NPs. On the contrary, SiO_2_NPs did not induce cytotoxic effects in this cell model. 

Intracellular GSH level changes (decreases) were also observed at both time-points (24 and 48 h) in A549 cells for all tested compounds (Cd-SiO_2_NPs, CdCl_2_, and SiO_2_NPs) suggesting an induction of oxidative stress. Notably, GSH level was the only altered parameter following SiO_2_NP exposure. 

Clonogenic assay, used to evaluate the effects induced after prolonged exposure (10 days), showed the ability of both Cd-SiO_2_NPs and CdCl_2_ (at the lowest tested dose of 0.05 *μ*g/mL) to drastically inhibit A549 cell proliferation, while, once again, SiO_2_NPs were avoided of any effect. 

In relation to cadmium toxicity, an extensive database is available on CdCl_2_-induced pneumotoxicant effects by *in vivo* and *in vitro* models [22]. Mechanistically, Cd, at the cellular level, has been shown to cause oxidative stress by depletion of endogenous antioxidants such as glutathione that is associated with mitochondrial damage and induction of apoptosis [8, 34]. A recent *in vitro* study underlined that low-dose cadmium triggers apoptosis rather than outright necrosis [35]. Indeed CdCl_2_ concentrations used in proximal tubule (PT) cell culture model to induce apoptosis ranged from 2 to 10 *μ*g/mL (corresponding to 10–50 *μ*M) [37]. For instance, these values are of similar magnitude as the threshold level of 50 *μ*g/g kidney tissue for the development of signs of kidney dysfunction and PT damage indicated in *in vivo* experimental and human studies of chronic Cd^2+^ [38, 39]. Involvement of caspase-3 has been described in several animal models of chronic Cd^2+^ nephrotoxicity [40, 41].

Apoptosis (5-fold higher than control) was also observed in cultures, of rat lung epithelial cell line, exposed for 48 h to 20 *μ*M CdCl_2_ [42], and was preceded by the up-regulation of oxidant stress genes (glutathione S-transferase-alpha, gamma-glutamylcysteine synthetase, and metallothionein-1), activation of redox sensitive transcription factors (AP-1 and NF-*κ*B), and changes in various forms of glutathione (reduced, oxidized, and protein-bound); thus, altogether, these features suggesting a key role played by the reactive oxygen species.

Our findings indeed evidenced, after both Cd-SiO_2_NPs and CdCl_2_, GSH depletion and activation of caspase-3 which is a critical executioner of apoptosis, as it is either partially or totally responsible for the proteolytic cleavage of many key proteins [43].

The observed cytotoxic effects induced by Cd-SiO_2_NPs after short-and long-term exposure suggest a crucial role of the cadmium moiety in the biological response to Cd-SiO_2_NPs although it seems unlikely that the changes produced by Cd-SiO_2_NPs merely reflected the action of cadmium ions released from nanoparticles. Indeed, chemical experiments with Cd-SiO_2_NPs have demonstrated limited release of cadmium ions from the nanoparticles dispersed in medium culture, the maximum metal release being ca. 28% over a 10-day period. 

In addition, the tendency to form aggregates and agglomerates of these doped NPs [29] may also have contributed in triggering the described Cd-SiO_2_NP effects: the DLS data demonstrated an agglomeration and aggregation extent of Cd-SiO_2_NPs (about 350 nm) greater than that measured for SiO_2_NPs (about 120 nm). Whether those agglomerated particles retain toxic properties of the individual nanoparticles or are capable of subsequently is a critical question [44].

An additional hypothesis may be related to the nano dimension of the material investigated. In our *in vitro* experiments, the nano dimension may have facilitated the cell concentration and thus toxicity of the administered cadmium. A “Trojan horse”-type mechanism involving silica nanoparticles as effective carriers for the cellular uptake of toxic metals has been described [45]. In the same type of lung cells used in this study, exposure to SiO_2_NPs doped with metals such as iron, manganese, cobalt, or titanium was shown to generate higher concentrations of reactive oxygen species and induce more severe oxidative stress compared to equivalent amounts of the respective metal ions [46]. In our study, incorporation of Cd into SiO_2_NPs may have increased the metal dose delivered to target cells although no specific data supporting this process are presently available.

With regard to SiO_2_NPs, no cytotoxic effects were observed for all concentrations tested and for all exposure times (acute or chronic), with the only exception for the observed GSH depletion after 48 h treatment. Several recent literature data are consistent with the present findings: SiO_2_NPs penetrated A549 cells and did not cause significant toxic effects at the molecular and cellular levels below 100 *μ*g/mL [47]; it induced low cytotoxicity at concentrations up to 200 *μ*g/mL [48], and generated oxidative stress reflected by reduction of GSH levels [16] or oxidant generation [49]. On the other hand, other investigations indicated A549 cell viability decreases after SiO_2_NPs exposure down to 100 *μ*g/mL [16], as well as a proinflammatory response triggered by SiO_2_NPs without blocking cell proliferation or causing cell death in A549 cells [50]. 

For comparison, our previous *in vivo* results indicated early and persistent lung damage after i.t. instillation of Cd-SiO_2_NPs in terms of enhanced apoptotic phenomena followed by a significant increase of proliferating cells [29], as well as pulmonary inflammation and fibrosis in rats evidenced by a wide-spread immunoreactivity of both cytokines/chemokines and collagen, respectively. The effects were detectable at the earliest time point, 24 h, and persisted until the 30th day post exposure. Similar pattern of toxic insult was also revealed after i.t. instillation of equivalent amount of CdCl_2_, although it was less marked than Cd-SiO_2_NP treatment. The dose of CdCl_2_ per animal was 400 *μ*g (2.1 *μ*mol) *≡* 247 *μ*g Cd. Cd-SiO_2_NPs and CdCl_2_ also showed the capacity to cause long-lasting oxidative stress by increasing the tissue F_2_-isoprostane levels and pulmonary SOD1, COX-2, and iNOS expressions [30].

On the contrary, no changes involving these markers were observed in animals treated with SiO_2_NPs. 

Altogether, the *in vivo* results showed a higher Cd-SiO_2_NPs reactivity (regardless of whether form type is present: original, agglomerate, or with sorbed material at NP surface) than SiO_2_NPs and CdCl_2_ in the lung tissue.

Both *in vitro* and *in vivo* findings pointed out that Cd-SiO_2_NP exposure produces a complex and multicomponent insult leading to an exacerbated toxicity response compared to the toxic pattern caused by CdCl_2_ treatment and essentially much more than SiO_2_NPs.

## 5. Conclusions

*In vitro* experiments in pulmonary cells have provided effective means of screening and ranking the tested materials (Cd-SiO_2_NPs > CdCl_2_ > SiO_2_NPs) using multiple toxicological endpoints (i.e., mitochondrial and membrane alterations, induction of apoptosis, inhibition of growth and proliferation, and intracellular GSH depletion). Coherently, the *in vivo* results have systematically characterized the tissue damage evidenced by lung parenchyma injury and fibrosis, apoptotic phenomena, the occurrence of inflammation, and pulmonary oxidative stress in rats. The *in vivo* targeted tests have complemented and addressed the *in vitro* findings to ensure the adequate evaluation of nanoparticle hazard potential, also in terms of time of appearance and persistence of the toxicological features on living organism.

## Highlights

- Cd-SiO_2_NPs produced in vitro toxic effects after short- and long-term exposure;
- similar toxic profile was observed after CdCl_2_, the effect rank: Cd-SiO_2_NPs > CdCl_2_;
- SiO_2_NPs influenced oxidative stress pathway only;
- *In vitro* tests on lung cells provided effective means of ranking the tested materials.
